# Editorial: Women in extreme microbiology: 2022

**DOI:** 10.3389/fmicb.2023.1339335

**Published:** 2023-11-30

**Authors:** Birgit Sattler, Monica Sharma

**Affiliations:** ^1^Department of Ecology, University of Innsbruck, Innsbruck, Austria; ^2^Austrian Polar Research Institute, Vienna, Austria; ^3^Department of Biotechnology, School of Life Sciences, Babasaheb Bhimrao Ambedkar University, Lucknow, India

**Keywords:** gender disparity in STEM, microbial life in Sicilian gypsum, antimicrobial effects, hypersaline environments, benthic marine microbes, *Sphingomonas* sp.

In recognition of International Women's Day 2022, Frontiers in Microbiology presents the Women in Extreme Microbiology collection, paying tribute to the outstanding work of women in the field. Not long ago, extreme microbiology research was predominantly male, but luckily, the number of women pursuing higher education in this field is increasing.

In the broader STEM landscape, a gender gap persists, with UNESCO statistics indicating that only 30% of researchers worldwide are women. They remain a minority among doctoral students and researchers, occupying just 26% of full professorships. UNESCO stresses the importance of achieving gender equality in science for sustainable development.

This Research Topic offers a platform to highlight the contributions of female researchers and the diverse range of research in extreme microbiology across different geographic regions and biological aspects. It aims to celebrate the achievements of women in this field and address gender disparities in science.

The paper of Rizk and Magdy shows potential impacts of fungi being capable to meatabolize on stones. This comprehensive study explores the unique characteristics and ecological role of *Hortaea werneckii* isolate GPS5 in rocky environments. GPS5 exhibits distinctive yeast-like growth with diverse colors and structures, demonstrating halo- and thermotolerance, and optimal growth at 20% NaCl and 25°C. Enzymatic assays highlight its versatility, especially in solubilizing calcium carbonate under 20% NaCl conditions, suggesting a role in stone degradation. Molecular characterization places GPS5 within the *H. werneckii* clade, indicating its close relationship with hypersaline environment isolates, showcasing its unique adaptation. Remarkably, GPS5 thrives within the Great Pyramid of Giza, thanks to these characteristics and its calcium carbonate solubilization ability. This study underscores the ecological dynamics and potential impact of this fungal species on stone structures in unique environments, with implications for conserving and understanding stone structures in arid regions.

Antarctica becomes the stage for the study by Núñez-Montero et al., where Antarctic *Sphingomonas* sp. So64.6b's genomic characteristics are explored to understand its adaptation to extreme conditions. This strain has the potential to produce novel antibiotics as an adaptation to Antarctic poly-extreme conditions, addressing the antibiotic crisis. Genomic exploration unveiled Biosynthetic Gene Clusters (BGCs) and unique adaptation-related genes, paving the way for potential antibiotic discoveries. Comparative genome analysis showed a strong link with the closest genus, *Sphingomonas alpina*, but significant genomic distinctions related to pollutant degradation. So64.6b harbors six BGCs, three of which show limited or no similarity to known clusters, potentially housing novel antibiotics. Phylogenetic analysis of a common BGC suggest its evolution within the *Sphingomonas* genus in specific ecosystems. Comparative genomics showed that *Sphingomonas* isolates from extreme environments possess more predicted BGCs and a higher genetic content dedicated to BGCs than mesophilic. Additionally, unique resistance genes were identified within the Antarctic strain, improving our understanding of antibiotic production in extreme environments.

Moving to northern Chile's Atacama Desert coastline, microbial communities' response to trace metals and anthropogenic coastal impacts is examined by Zárate et al. using environmental DNA and RNA (eDNA/eRNA) to assess ecological quality. The research focuses on sediment deposition in northern Chile, specifically examining the effects of Cu and Fe on benthic marine microbes through genetic analysis. The results show significant variations in microbial communities, with habitat influencing co-occurrence networks. Discriminant analysis identifies key taxa like *Beggiatoaceae, Carnobacteriaceae*, and *Nitrosococcaceae* in Off Loa, and *Enterobacteriaceae, Corynebacteriaceae, Latescibacteraceae*, and *Clostridiaceae* in Mejillones Bay as responsible for differences in eDNA and eRNA between zones. Multivariate analysis demonstrates a connection between microbial assemblages and Cu and Fe fractions, especially in the Bay. Predicted functional structures reveal the role of transporters and DNA repair in responding to metals and environmental factors. The study also identifies active taxa associated with anthropogenic impacts, potentially carrying antibiotic resistance. In summary, eDNA and eRNA improve our understanding of microbial communities, aiding in marine environment management.

In Southern Sicily, endolithic colonization within gypsum is investigated by Němečková et al., revealing cyanobacteria as the predominant microorganisms. They explore the diversity and distribution of phototrophic microorganisms across Sicilian gypsum sites. Cyanobacteria, including *Chroococcidiopsis* sp., *Gloeocapsopsis pleurocapsoides, Gloeocapsa compacta, Nostoc* sp., and orange-pigmented green microalgae from the *Stephanospherinia* clade, prevail in these environments. The study employs single-cell and filament sequencing, 16S rRNA amplicon metagenomics, and Raman spectroscopy to unveil the phylogenetic diversity and taxonomic identification of these cyanobacteria, highlighting distinct pigment zones within the gypsum. Carotenoids are the primary pigments, with additional markers like gloeocapsin and scytonemin near the surface. This research deepens our understanding of phototrophic microorganism diversity in Southern Sicilian gypsum, emphasizing the complex nature of endolithic ecosystems and their responsiveness to different gypsum types. It enhances our knowledge of the overall bioreceptivity of such environments.

Lastly, Arcadi et al. explore shallow hydrothermal vents at Levante Bay on Volcano Island as natural laboratories to study the effects of acidification on biota. These vents release CO_2_, lowering pH levels. Next Generation Sequencing is employed to study microbial communities in water and sediment samples, revealing significant taxonomic changes, particularly in sediments. This research deepens our understanding of microbial communities in unique marine environments and their response to environmental forces, emphasizing substantial alterations in microbial community structure and their potential as acidification indicators, affecting biogeochemical balance in stressed ecosystems. In summary, this study underscores the value of microbial communities as acidification indicators in distinctive marine environments.

This compilation holds paramount importance due to its multifaceted contributions to our scientific knowledge and practical applications. It provides a deeper understanding of microbial life in extreme and unique environments, expanding our comprehension of the Earth's biodiversity and the extraordinary adaptability of microorganisms to challenging conditions. Ultimately, these studies contribute to our broader scientific and environmental goals, helping us to increase the visibility of females and to take informed decisions for the conservation and sustainable management of our planet's diverse habitats.

## Author contributions

BS: Conceptualization, Validation, Writing – original draft, Writing – review & editing. MS: Conceptualization, Writing – review & editing.

